# Genome-Scale NAD(H/^+^) Availability Patterns as a Differentiating Feature between *Saccharomyces cerevisiae* and *Scheffersomyces stipitis* in Relation to Fermentative Metabolism

**DOI:** 10.1371/journal.pone.0087494

**Published:** 2014-01-29

**Authors:** Alejandro Acevedo, German Aroca, Raul Conejeros

**Affiliations:** 1 Escuela de Ingeniería Bioquímica/Pontificia Universidad Católica de Valparaíso, Valparaíso, Chile; 2 Bioenercel S.A., Barrio Universitario, Concepción, Chile; Texas A&M University, United States of America

## Abstract

*Scheffersomyces stipitis* is a yeast able to ferment pentoses to ethanol, unlike *Saccharomyces cerevisiae*, it does not present the so-called overflow phenomenon. Metabolic features characterizing the presence or not of this phenomenon have not been fully elucidated. This work proposes that genome-scale metabolic response to variations in NAD(H/^+^) availability characterizes fermentative behavior in both yeasts. Thus, differentiating features in *S. stipitis* and *S. cerevisiae* were determined analyzing growth sensitivity response to changes in available reducing capacity in relation to ethanol production capacity and overall metabolic flux span. Using genome-scale constraint-based metabolic models, phenotypic phase planes and shadow price analyses, an excess of available reducing capacity for growth was found in *S. cerevisiae* at every metabolic phenotype where growth is limited by oxygen uptake, while in *S. stipitis* this was observed only for a subset of those phenotypes. Moreover, by using flux variability analysis, an increased metabolic flux span was found in *S. cerevisiae* at growth limited by oxygen uptake, while in *S. stipitis* flux span was invariant. Therefore, each yeast can be characterized by a significantly different metabolic response and flux span when growth is limited by oxygen uptake, both features suggesting a higher metabolic flexibility in *S. cerevisiae*. By applying an optimization-based approach on the genome-scale models, three single reaction deletions were found to generate in *S. stipitis* the reducing capacity availability pattern found in *S. cerevisiae*, two of them correspond to reactions involved in the overflow phenomenon. These results show a close relationship between the growth sensitivity response given by the metabolic network and fermentative behavior.

## Introduction

Pentoses fermenting yeasts are important due to their relevance in the production of second generation biofuels, since xylose is the main pentose found in hemicelluloses and its fermentation to ethanol is key to obtain economically viable processes [1]–[3]. *Scheffersomyces stipitis* is a yeast well known for its ability to ferment xylose to ethanol and has been a matter of research in a broad number of studies, including two recently published genome-scale metabolic reconstructions [4], [5]. *S. stipitis* does not present the so-called Crabtree effect or overflow phenomenon [6], [7] like *Saccharomyces cerevisiae* does. This phenomenon refers to the occurrence of fermentative metabolism along with aerobic respiration (respiro-fermentative metabolism) triggered by high glucose uptake rates [8], [9]. This effect is observed in aerobic chemostat culture by a shift from fully respiratory metabolism towards fermentative metabolism at increased dilution rates [10], [11]. In the case of *S. stipitis*, it induces ethanol production only when growth is limited by oxygen availability [12], [13], that is within a narrow range of oxygen concentration in the culture. *S. stipitis*'s strong dependency between ethanol production and oxygen availability, regardless of carbon source availability levels, has been widely described in literature [14]–[18]. However, in both yeasts a decrease in oxygen uptake rate is associated to fermentative metabolism, which in the case of *S. cerevisiae* is driven by the overflow phenomenon and in *S. stipitis* by the oxygen limiting conditions. *S. stipitis* presents the proton translocating NADH dehydrogenase complex I (NDH1) and an alternative oxidase (AOX) at the respiratory chain, both absent in *S. cerevisiae*. This determines in *S. stipitis* a higher respiratory capacity than *S. cerevisiae* [19]. Presence of AOX has been reported as highly prevalent in Crabtree negative yeast species [20].

Although fermentative metabolism has been widely studied in yeast, overall metabolic properties characterizing the occurrence of the overflow phenomenon are not fully understood [9]. Elucidating which features differentiate a yeast such as *S. stipitis* from *S. cerevisiae* may help the understanding of fermentative behavior and the metabolic engineering of ethanol producing strains. Fermentative metabolism is affected by NAD(H/^+^) cofactors and there is evidence showing that high NADH availability promotes fermentation. This is supported by a number of reports indicating that cofactor manipulation is a useful tool for improving fermentation performance [21]. It has been shown in *E. coli* that an increment in NADH availability induces fermentation by stimulating pathways which are normally inactive under aerobic conditions [22]. Vemuri et al. [23] also demonstrated in *S. cerevisiae* that the oxidation capacity of NADH is directly related to the occurrence of the overflow phenomenon, they were able to reduce it in a great extent by introducing a heterologous alternative oxidase (AOX) at the mitochondrion. Furthermore, Hou et al. [24] using a *S. cerevisiae* strain disabled for formate anabolism, and modified to overexpress the native NAD dependent formate dehydrogenase, were able to induce fermentation by increasing intracellular NADH concentration via formate supplementation.

Regarding cofactor usage in *S. stipitis* the first two steps of xylose consumption produces an stoichiometric imbalance yielding a positive NADH balance. *S. stipitis* may prevent this NADH excess by converting NADH to NADPH through a bypass at the tricarboxylic acids cycle (TCA) [4], [25]. This cofactor imbalance may not be avoided in recombinant xylose consuming *S. cerevisiae* strains, often leading to xylitol accumulation [26]. Also, the expression of the alternative oxidase has been shown to be induced under oxygen limited conditions with xylose consumption, serving as an electron sink [19]. Furthermore, *S. stipitis* may be able to use the arabinose assimilation pathway backwards, oxidizing NADH and producing polyols. Accumulation of polyols in *S. stipitis* is several fold greater than in *S. cerevisiae* in aerobic batch culture (317 fold in arabitol, 46 fold in ribitol) [18]. Diano et al. [27] have shown that in *Aspergillus niger* polyols are synthesized under oxygen limiting conditions, where they play a role in the balance of redox metabolism. Hence, evidence suggests that the metabolism of *S. stipitis* efficiently recycles NAD(H/^+^) cofactors, so that ethanol production under oxygen limiting conditions may be related to a compromise in the use of NADH for growth. On the other hand, NAD(H/^+^) availability is related to the flexibility of the flux network. Regarding this, using genome-scale metabolic modeling, Ghosh et al.[28] studied the effect of the cofactor imbalanced pathways in *S. cerevisiae*, finding that cofactor imbalance increases the range of flux variability (flux span). Thus, an excess of NADH can lead to an increase in metabolic flexibility, allowing the induction of fermentative metabolism. Overall, the availability levels of NADH in both yeasts could lead to a metabolic response that determines whether NADH is used for growth or not. Therefore, growth sensitivity response to changes in NADH metabolic availability might be different in each yeast and may serve characterizing fermentative behavior.

A helpful way to study overall metabolic properties is by using genome-scale metabolic reconstructions [29]. These are curated and empirically validated knowledge bases in which all known chemical reactions of an organism are detailed and cataloged [30]. For predicting phenotypes from the genome-scale metabolic reconstructions, Flux balance analysis (FBA) [31], [32] is used. FBA uses linear optimization principles where an objective function (for example, biomass production) is maximized subject to the constraints imposed by the metabolic network and metabolite uptake rates. This method is the basis of the constraint-based analyses [30], [33] and it is used to find the flux distribution of the metabolic network. In FBA, cofactor availability can be studied via the sensitivity analysis of the linear optimization problem involved in the mathematical metabolic model, also known as shadow price analysis [34], [35]. Shadow price refers to the sensitivity of and objective function (biomass production) to differential changes on a resource availability, e.g. it indicates how much the biomass production will increase or decrease with differential change in a metabolite availability [35], [36], thus it allows determining whether a metabolite is in excess or limiting for biomass production. By using previously reported data [37] of the change in the intracellular metabolite abundance at different growth rates and relating it to the FBA of a genome-scale metabolic model of *S. cerevisiae*, Reznik et al. [35] have recently demonstrated that shadow price analysis can be used as an index of the response of cellular growth to variations in metabolic resource availability. Thus, shadow prices determine the resource availability pattern showing if it is limiting or is in excess for biomass production.

Despite the amount of reports concerning fermentation in yeast, there are a few studies on how NAD(H/^+^) availability affects fermentative behavior considering the genome-scale metabolic network [4], [21], [28] and at present there is only one report showing a comparative systems biology study between Crabtree-positive and negative yeasts [18].

This work aims to determine the differentiating metabolic features between *S. stipitis* and *S. cerevisiae* regarding NAD(H/^+^) availability patterns in relation to ethanol production capacity and overall metabolic flux span. By considering the difference between NADH and NAD shadow prices as an index for available reducing capacity, which defines the ability of the flux-carrying pathways for supplying the NADH demand for growth, NAD(H/^+^) availability was studied. Ethanol production capacity was analyzed using production envelope analysis to characterize the relationship between growth and the production rate of ethanol. Flux variability analysis [38] was used to evaluate overall metabolic flux span as an index of metabolic flexibility. Phenotypic phase planes analysis [36] was performed to characterize different metabolic phenotypes in a range of growth conditions considering different uptake rates, these included fully respiratory growth and limited by oxygen uptake rate. Using production envelope analysis and experimental results from literature [18], metabolic phenotypes at exponential growth were mapped, showing the characteristic coupling between ethanol production and growth given by the overflow phenomenon in *S. cerevisiae*. From shadow price and phenotypic phase planes analyses differences in reducing capacity availability patterns were found. In *S. cerevisiae* an excess in available reducing capacity for growth was observed at every metabolic phenotype where growth is limited by oxygen uptake rate, while in *S. stipitis* this was found only at some of those phenotypes. Thus, phenotypes at exponential growth mapped in production envelope analysis are allocated in phenotypic phases of excess and limitation of available reducing capacity, for *S. cerevisiae* and *S. stipitis* respectively. Flux variability analysis showed an increased metabolic flux span in *S. cerevisiae* when growth is limited by oxygen uptake rate, while in *S. stipitis* flux span was invariant. This increased flux span along with the excess of available reducing capacity suggest high flexibility of the metabolic network of *S. cerevisiae*, allowing the overflow phenomenon. Furthermore, the relationship between these differentiating features to a metabolic function: the overflow phenomenon, was studied by seeking modifications able to generate excess of available reducing capacity in all phenotypes where growth is limited by oxygen uptake rate, and verifying their involvement in the phenomenon. Thus, deletions in *S. stipitis* generating the pattern of excess found in *S. cerevisiae* were searched for. By using an optimization-based approach it was found that the deletion of reactions in *S. stipitis*, which are negatively regulated during overflow phenomenon in *S. cerevisiae*, allowed the replication of the sought reducing capacity availability pattern.

Hence, the relevance of the results of this work is that they allow establishing a close relationship between growth sensitivity response, regarding available reducing capacity, and fermentative behavior.

## Results

### Characterization of ethanol producing capacity

**Figure 1:**
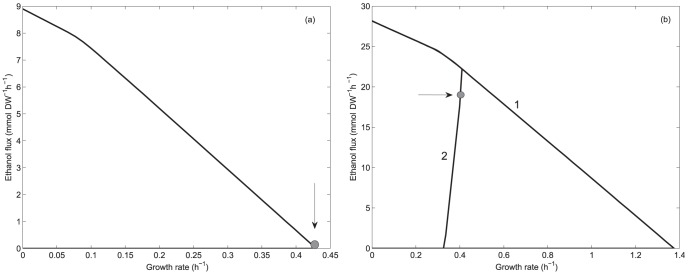
Envelopes for ethanol production and growth rate. (a): Envelope for *S. stipitis*. Glucose uptake rate of 4.45 mmol gDW^−1^ h^−1^ and unbounded oxygen uptake rate were considered. (b): Envelopes for *S. cerevisiae*. Glucose uptake rate of 14.08 mmol gDW^−1^ h^−1^ considering unbounded oxygen uptake rate (envelope 1), and an upper bound of 2.8 mmol gDW^−1^ h^−1^ (envelope 2). Arrows indicate data reported by Papini et al. [18].

Figures 1a and b show the production envelopes describing the relationship between ethanol production and growth rate for both yeasts. As constraint-based models describe both biomass and metabolite production rates, favoring the latter could have a negative effect on biomass production. Production envelopes characterize this flux relationship, thus two situations can be observed: 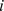) if every time one of the fluxes is enhanced, the other is impaired, then a trade-off exists. 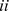) if both fluxes can be increased simultaneously, then it means they are coupled. Envelopes were calculated using data reported by Papini et al. [18] at exponential growth in aerobic batch cultures. For *S. stipitis* (Figure 1a) a glucose uptake rate of 4.45 mmol gDW^−1^ h^−1^ (26.7 C-mmol gDW^−1^ h^−1^) and unbounded oxygen uptake rate were considered. As Figure 1a shows, a maximum specific growth rate of 0.429 h^−1^ is predicted by the model, which is close to the value reported by Papini et al. [18]; 0.47 h^−1^, at aerobic conditions where no ethanol production is observed. This envelope shows that in *S. stipitis* there is no coupling between growth and ethanol production at aerobic batch conditions. For *S. cerevisiae*, envelope 1 in Figure 1b corresponds to the one calculated considering a glucose uptake rate of 14.08 mmol gDW^−1^ h^−1^ (84.5 C-mmol gDW^−1^ h^−1^) and unbounded oxygen uptake rate, while envelope 2 shows the one calculated considering an upper bound of 2.8 mmol gDW^−1^ h^−1^ for oxygen uptake rate, fitting the envelope close to the experimental values reported by Papini et al. [18]; a specific growth rate of 0.40 h^−1^ and a specific ethanol productivity of 19.90 mmol gDW^−1^ h^−1^ (calculated from their data). As it can be observed, in contrast to *S. stipitis*, ethanol production and growth rate are coupled in *S. cerevisiae*, accounting for the presence of the overflow phenomenon. Arrows in Figure 1a and b indicate metabolic phenotypes at exponential growth in aerobic batch considering data reported by Papini et al. [18]. To characterize these phenotypes regarding their NAD(H/^+^) availability patterns, phenotypic phase planes (PhPPs) and shadow price analyses were carried out for each yeast.

### Phenotypic phase planes analysis

**Figure 2:**
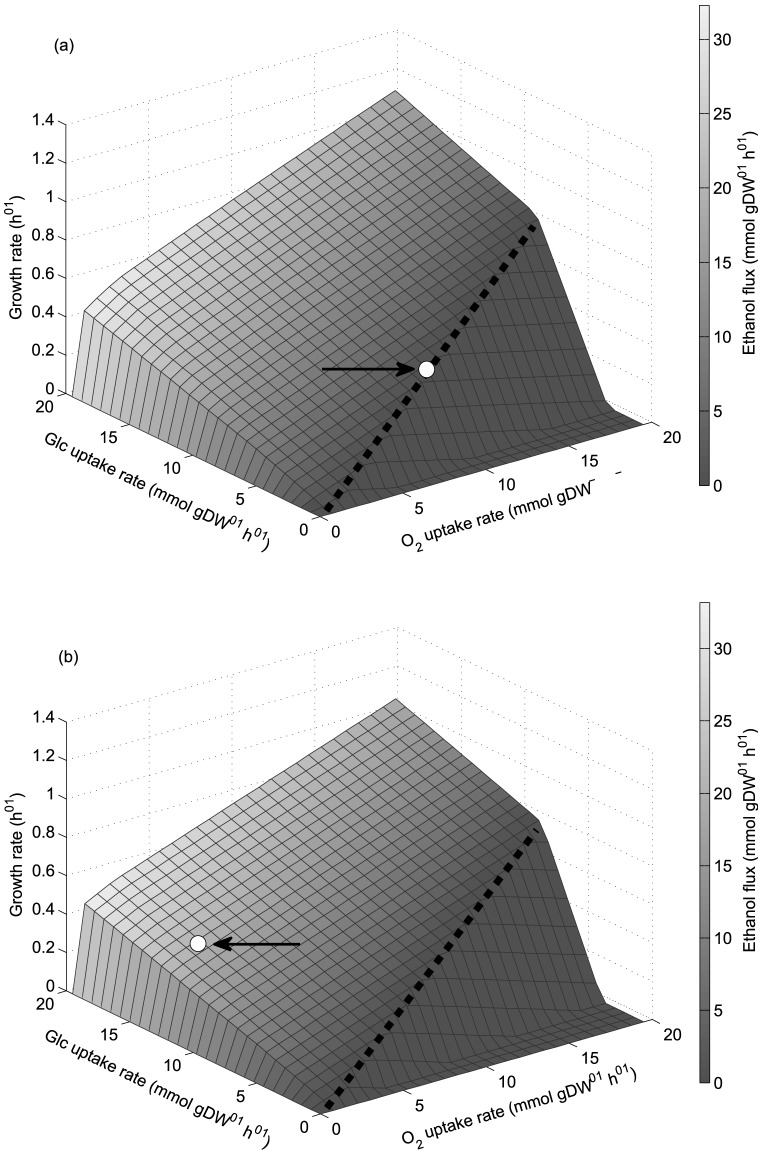
Phenotypic phase planes characterizing growth and ethanol production as a function of uptake rates. (a): *S. stipitis*. (b): *S. cerevisiae*. Arrows and white dots indicate data reported by Papini et al. [18]. Dashed black line: line of optimality. Glc: glucose.

PhPPs analysis was performed characterizing growth rate as a function of glucose and oxygen uptake rates. Figures 2a and b show the PhPPs for *S. stipitis* and *S. cerevisiae*. Each phase plane represents a qualitatively distinct phenotype having different optimal use of the flux network [36]. The dashed black line corresponds to the line of optimality, here oxygen and glucose uptake rates are in the exact ratio to allow maximal biomass yield, i.e., a fully respiratory metabolism. To the left of this line growth is limited by oxygen uptake rate, corresponding to phases where ethanol production occurs, and to the right it is limited by glucose uptake rate. Data obtained by Papini et al. [18] at exponential growth in aerobic batch are allocated over the planes, arrows and white dots indicate their positions (Figures 2a and b). As expected from the envelope analyses (Figure 1a and b), *S. stipitis'*s metabolism is on the line of optimality, indicating a fully respiratory metabolism, while in *S. cerevisiae*, the occurrence of the overflow phenomenon, allocates its metabolism in a state characterized by high glucose uptake rate and growth limited by oxygen uptake rate.

### Shadow price analysis

**Figure 3:**
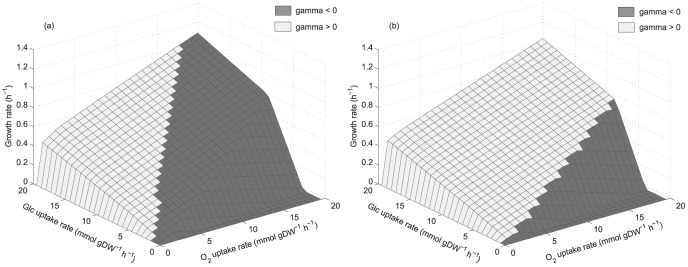
Phenotypic phase planes showing reducing capacity availability patterns. (a): *S. stipitis*. (b): *S. cerevisiae*. 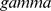: 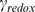, shadow price for available reducing capacity (Eq. 2). Phase planes are shaded showing if 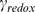 is positive or negative. Glc: glucose.

Once the PhPPs for each yeast has been obtained, shadow price analysis was performed to study the cellular growth response to changes in the available reducing capacity, which is defined here as the difference between NADH and NAD shadow prices. A difference between these shadow prices indicates that their requirement for biomass production is different, for example a net demand for NADH may lead to an excess of NAD. Thus, we define reducing capacity as the ability to satisfy this NADH demand. On the other hand, if there is a net demand of NAD, NADH will be in excess, then an oxidizing capacity for NADH is required. This is defined as an excess of reducing capacity. Consequently, the difference between NADH and NAD shadow prices can be defined as the availability of the reducing capacity with respect to its demand for growth, and is an indicator showing if it is in excess or limiting for growth, in short: available reducing capacity. Whether the available reducing capacity is in excess or not is shown by the sign of the resulting shadow price; negative for limiting and positive for excess (see Methods section for details). The excess or limitation at different phenotypic phases determines the availability pattern. Figure 3a and b show the reducing capacity availability patterns, the PhPPs are shaded depending whether the value of the shadow price for available reducing capacity (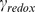, Eq. 2 in Methods) is positive or negative. For *S. stipitis* (Figure 3a), 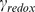 is positive in a region where growth is limited by oxygen uptake rate, this sensitivity response means that a decrease in the available reducing capacity increases growth rate, so that within this region there is an excess of this resource for biomass production. For *S. cerevisiae*, 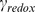 is greater than zero in all phase planes where growth is limited by oxygen uptake rate (Figure 3b). It has to be noted that from this analysis an excess of available reducing capacity does not imply it accumulation. Therefore, where shadow price is positive, available reducing capacity exceeds the optimal demand for growth and alternative routes such as fermentative pathways may be used to balance redox metabolism. Hence, shadow price analysis shows that, at growth limited by oxygen uptake rate, each yeast presents a different metabolic response to changes in the available reducing capacity, thus a characteristic reducing capacity availability pattern (excess in a wide range of phenotypes) was found in *S. cerevisiae*. Moreover, comparing Figure 3 with Figure 2, it can be noted that, metabolic phenotypes at exponential growth rate for *S. stipitis* and *S. cerevisiae* from data reported by Papini et al. [18], allocate in regions where available reducing capacity is growth-limiting or in excess respectively.

### Flux variability analysis

**Figure 4:**
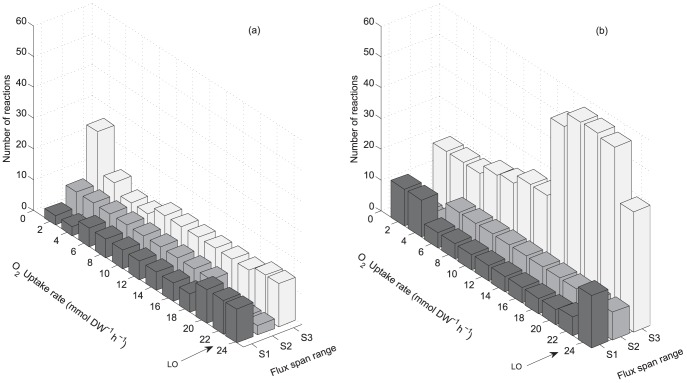
Frequency for different ranges of flux span at growth limited by oxygen uptake rate. (a): *S. stipitis*. (b): *S. cerevisiae*. S1: Flux span (Eq.3) greater than 10 mmol gDW^−1^h^−1^, S2: Flux span from 1 to 10 mmol gDW^−1^ h^−1^, S3: Flux span from 0.01 to 1 mmol gDW^−1^ h^−1^. LO: Line of optimality. Arrow indicates flux span at the line of optimality. Flux variability was calculated using glucose uptake rate of 10 mmol gDW^−1^ h^−1^.

Considering the differences found between the metabolisms of both yeasts regarding reducing capacity availability patterns (Figure 3a and b), overall metabolic flux span was analyzed in order to evaluate metabolic flexibility. The solution obtained from the FBA allows a range of flux values which yield the same optimal biomass production. This range corresponds to the flux span and was calculated via Flux Variability Analysis [38]. Figures 4a and b show the overall metabolic flux span for both yeasts at growth limited by oxygen uptake rate. Reactions were classified according to their flux span value. Three groups having different range of flux span where considered; from 0.01 to 1 mmol gDW^−1^ h^−1^, from 1 to 10 mmol gDW^−1^ h^−1^ and greater than 10 mmol gDW^−1^ h^−1^. Figure 4a and b does not to show the reactions with flux span less than 0.01 mmol gDW^−1^ h^−1^, thus only the more substantial changes are emphasized. In *S. cerevisiae* it was found that the number of reactions with a flux span between 0.01 to 1 mmol gDW^−1^ h^−1^ increases when growth is limited by oxygen uptake, this is shown in Figure 4b towards lower oxygen uptake rates. Figure 4a shows that for *S. stipitis* there is an almost invariant overall flux span. Therefore, the increased metabolic flux span found in *S. cerevisiae* could be associated with its characteristic reducing capacity availability pattern (Figure 3b), suggesting a relationship between metabolic flux span and cofactor availability.

Up to this point, two differentiating features were found for both yeasts in relation to properties associated to their genome-scale metabolic networks. These are the reducing capacity availability patterns and the metabolic flux span, both found at growth limited by oxygen uptake. As it will be seen, these differences may be relevant to their characteristic fermentative behavior.

### Reaction deletion analysis

Considering that the most relevant differentiating feature between these yeasts is the presence or not of the overflow phenomenon, the relationship between reducing capacity availability patterns and this metabolic function was studied. Therefore, modifications able to generate excess of available reducing capacity in all phenotypes where growth is limited by oxygen uptake rate were searched for, and then their involvement in the overflow phenomenon was subsequently verified. Thus, the aim was to find modifications to replicate in *S. stipitis*, the 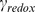 pattern found in *S. cerevisiae*. To achieve this, the strategy proposed in this work is the modification (reaction deletions) of the respiratory metabolism with the aim of forcing it to avoid the use of available reducing capacity for biomass production. Two approaches were used to find deletions: Firstly, an *a priori* approach which considered the modification of the mitochondrial NADH-consuming related pathways, with the purpose of decreasing respiratory capacity, expecting to generate the pattern found in *S. cerevisiae*. These modifications were: (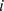) a double-deletion considering the NADH dehydrogenase complex I (NDH1) and the alternative oxidase (AOX) because of the absence of both in *S. cerevisiae*, and (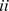) a deletion of the standard cytochrome oxidase (COX), because a significant ethanol production at high aeration conditions has been reported in a strain of *S. stipitis* having this single-deletion [39]. Secondly, a systemic optimization-based approach searching for deletions which may enhance ethanol flux was used.

#### *A priori* approach

**Figure 5:**
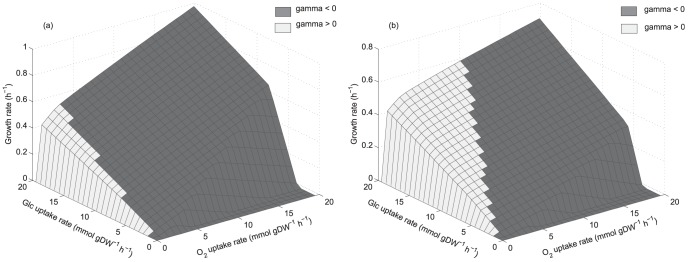
Phenotypic phase planes showing reducing capacity availability patterns for *S. stipitis* considering *a priori* deletions. (a): NDH1/AOX double-deletion. (b): COX single-deletion. 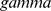: 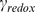, shadow price for available reducing capacity (Eq. 2). Phase planes are shaded showing where 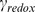 is positive or negative. Glc: glucose.

PhPPs analysis showed that by applying the NDH1/AOX double deletion and COX single deletion, 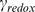 was positive only at low oxygen uptake rates, away from the optimality line (Figure 5a and b). In agreement with Freese et al. [39], whom reported high ethanol production at aerobic conditions in a COX single-knockout strain, PhPPs for COX deletion (Figure 5b) show that high growth rates are associated to excess of available reducing capacity and high ethanol flux. Therefore, the excess in available reducing capacity away from the optimality line remains after applying modifications which have been suggested to be relevant for respiratory and fermentative metabolism [20], [23], [39].

#### Optimization-based approach

**Figure 6:**
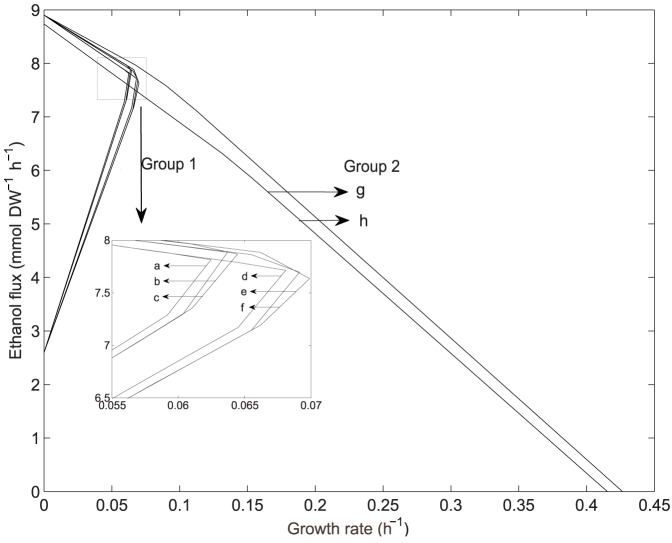
Production envelopes for *S. stipitis* having deletions found via the optimization-based approach. Group 1: Envelopes showing coupling between growth and ethanol production. Group 2: Envelopes showing non-coupling between growth and ethanol production. Arrows indicate envelope's corresponding group. Glucose uptake rate: 4.45 mmol gDW^−1^ h^−1^. Unbounded oxygen uptake rate was considered for all calculations. Deletions: (a) AOXIDASE/ATPSm/GLUD1, (b) AOXIDASE/CYOOm/GLUD1, (c) AOXIDASE/CYOR_u6m/GLUD1, (d) AOXIDASE/ATPSm, (e) AOXIDASE/CYOOm, (f) AOXIDASE/CYOR_u6m, (g) HEX1, (h) PGCD (see Table 1 for reaction names).

**Figure 7:**
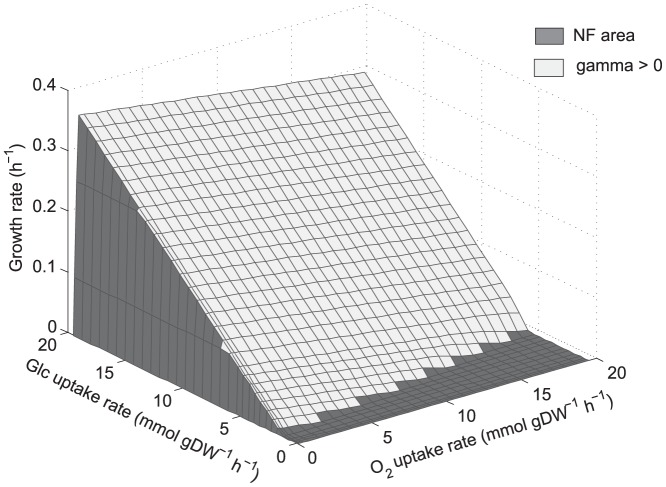
Phenotypic phase planes showing reducing capacity availability patterns for *S. stipitis* having triple-deletion (AOX/ATPSm/GLUD1). 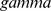
: 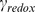, shadow price for available reducing capacity (Eq. 2). Phase planes are shaded showing 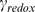 sign. NF: Not feasible. Glc: glucose.

**Figure 8:**
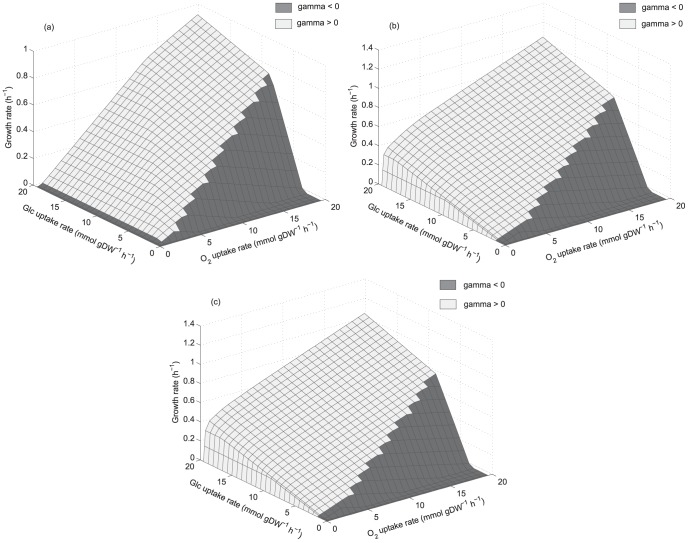
Phenotypic phase planes for *S. stipitis*'s modified phenotypes showing *S. cerevisiae*'s 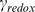 pattern. (a): HEX1 deletion, (b): GLUD1 deletion and (c): PGCD deletion. 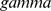: 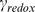, shadow price for available reducing capacity (Eq. 2). Phase planes are shaded showing if 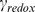 is positive or negative. Glc: glucose.

An optimization-based search for reaction deletions in *S. stipitis* which may enhance ethanol production and at the same time resemble the 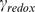 pattern of *S. cerevisiae* was carried out. Deletions found were divided in two groups; the first one characterized by coupled growth and ethanol production, these are listed in Table 1 and their production envelopes are shown in Figure 6 (group 1). As an example Figure 7 shows the 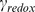 pattern for one phenotype of this group corresponding to a triple-deletion (AOX/ATPSm/GLUD1). In this case, 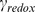 is positive throughout all the PhPPs, which means that there is always an excess of available reducing capacity, the same pattern was found in the rest of the group. Although it is possible to establish a link between excess of available reducing capacity and coupling between growth and ethanol production, deletions obtained for group 1 resulted in phenotypes showing growth rates always decreasing when increasing oxygen uptake rates for a given glucose uptake rate (Figure 7), which is very different from the PhPPs of *S. cerevisiae* (Figure 3b). As the obtained deletions did not result in the expected PhPP and 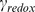 patterns, deletions allowing higher specific growth rates were calculated. By constraining for higher growth rates in the optimization-based approach, phenotypes showing non-coupled growth and ethanol production were obtained. This procedure yielded two deletions showing the same 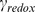 pattern as the one observed in *S. cerevisiae* and they are shown in group 2 of Table 1, corresponding to hexokinase 1 (HEX1) and phosphoglycerate dehydrogenase (PGCD). Production envelopes of each one are shown in Figure 6 labeled as group 2 and 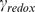 patterns are shown in Figure 8a and b. By using the modified model (see methods section) one extra single deletion was found, corresponding to the glutamate dehydrogenase NADH depending (GLUD1) reaction (Table 1), its corresponding PhPPs and 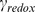 pattern are shown in Figure 8c. Overall, three single deletions were able to generate in *S. stipitis* the 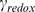 pattern found in *S. cerevisiae*. Therefore, available reducing capacity patterns of *S. cerevisiae* can be replicated in *S. stipitis*. Two of these reactions, HEX1 and PGCD, are directly involved in the overflow phenomenon [11], [18].

**Table 1:** Deletions found using the systemic approach.

Group 1: Phenotypes with coupled growth and ethanol production		
Deleted reactions	Enzyme	Related metabolism
AOXIDASE	Alternative oxidase	
ATPSm	mitochondrial ATP synthase	Oxidative phosphorylation/
GLUD1	Glutamate dehydrogenase (NAD)	Nitrogen metabolism
AOXIDASE	Alternative oxidase	
CYOOm	cytochrome c oxidase, mithocondrial	Oxidative phosphorylation/
GLUD1	Glutamate dehydrogenase (NAD)	Nitrogen metabolism
AOXIDASE	Alternative oxidase	
CYOR_u6m	Ubiquinol-6 cytochrome c reductase	Oxidative phosphorylation
GLUD1	Glutamate dehydrogenase (NAD)	
AOXIDASE	Alternative oxidase	
ATPSm	ATP synthase, mitochondrial	Oxidative phosphorylation
AOXIDASE	Alternative oxidase	
CYOOm	cytochrome c oxidase, mitochondrial	Oxidative phosphorylation
AOXIDASE	Alternative oxidase	
CYOR_u6m	Ubiquinol-6 cytochrome c reductase	Oxidative phosphorylation

Group 2: Phenotypes with non-coupled growth and ethanol production		
Deleted reactions	Enzyme	Related metabolism
HEX1	Hexokinase 1	Glycolysis
		
PGCD	Phophoglycerate dehydrogenase	Glycine, serine and
		threonine metabolism
**Deletion found using the modified model**		
Deleted reactions	Enzyme	Related metabolism
GLUD1	Glutamate dehydrogenase	Nitrogen metabolism
	NADH dependent	

## Discussion

From production envelope analysis of biomass and ethanol fluxes, coupling for *S. cerevisiae* and non-coupling for *S. stipitis* was observed. This is because the overflow phenomenon which leads to fermentation in *S. cerevisiae* is absent in *S. stipitis* [11], [18], [40]. For the phenotypic phase planes of *S. cerevisiae*, shadow price for available reducing capacity (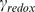) was in excess in all phenotypic phases where growth is limited by oxygen uptake rate (Figure 3b), this excess may favor the production of ethanol in a wide range of metabolic phenotypes. This is in accordance with reports showing a relationship between NADH availability and fermentative behavior, showing that fermentation can be favored by high NADH availability [22]–[24]. In the case of *S. stipitis*, 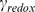 pattern shows that at growth limited by oxygen uptake, although ethanol is produced, its metabolism would not favor fermentation to ethanol by allowing a more efficient use of available reducing capacity for growth than *S. cerevisiae*. This agrees with studies showing a relevant efficiency in NAD(H/^+^) cofactor recycling in *S. stipitis*, by using pathways such as: NADH to NADPH conversion through a by-pass at the TCA cycle, the alternative oxidase at the respiratory chain and polyol synthesis [4], [18], [19], [25]. Therefore, each yeast shows a different metabolic network response to available reducing capacity which may be related to their characteristic fermentative metabolism.

Flux variability was evaluated for both yeasts in phenotypic phases where growth is limited by oxygen uptake rate. A large increase in overall flux span was found in *S. cerevisiae*, while in *S. stipitis* flux span was almost invariant (Figure 4a and b). Then, two differentiating features between both yeasts were identified in this work; the characteristic 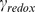 patterns and the overall metabolic flux span. A relationship between NAD(H/^+^) cofactors availability and overall flux span has been established by Ghosh et al. [28]. Using a genome-scale metabolic model, they studied the effect of the cofactor imbalance caused by the insertion of xylose assimilation pathways in *S. cerevisiae*, finding that the modified model (cofactor imbalanced) presents larger overall metabolic flux span than the wildtype, showing that cofactor imbalance was responsible for this increase in flux span. In this work, the characteristic excess of available reducing capacity found in *S. cerevisiae*, as in the case of the cofactor imbalanced model studied by Ghosh et al. [28], is associated to an increased overall flux span. Therefore, it is proposed that when available reducing capacity is in excess for biomass production, like in *S. cerevisiae* at all phenotypes with growth limited by oxygen uptake, the number of feasible alternate optimal states increases, thus increasing the metabolic network flexibility. On the other hand, available reducing capacity in *S. stipitis* is not always in excess, and metabolic flexibility is not increased. Reznik et al.[35], reports that metabolites with large negative shadow price values show little variation in concentration after uptake rates changes (environmental perturbations), and the more positive they are, the more likely to present larger variations. Thus, at growth limited by oxygen uptake, higher variations in NADH concentration are expected in *S. cerevisiae* than in *S. stipitis*. This along with the increased flux span of *S. cerevisiae* may provide the metabolic flexibility to allow the overflow phenomenon.

Reaction deletions which may replicate in *S. stipitis* the 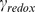 patterns found in *S. cerevisiae* were found. COX single deletion resulted in a phenotype with high growth rates associated with high ethanol flux and excess of available reducing capacity (Figure 5b). This is in accordance with the findings of Freese et al.[39] reporting significant ethanol production at aerobic conditions in *S. stipitis* when COX gene is knocked-out. From the optimization-based approach three single-deletions were able to generate the 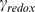 pattern of *S. cerevisiae* (Figure 8a, b and c); these were HXK1, PGCD and GLUD1. It has been reported that both the carbon flux through PGCD, which leads to serine synthesis, and the expression of HXK1 decrease in a great extent when overflow phenomenon is induced [11], [18]. This suggests a link between this phenomenon and the sensitivity response regarding available reducing capacity. For the deletion of GLUD1, which catalyzes the first reaction in the above mentioned bypass at the TCA cycle, there are no reports showing if GLUD1 is directly involved in the induction of the overflow phenomenon.

In conclusion, the results of this research allow relating metabolic sensitivity response to changes in available reducing capacity, to fermentative behavior. It was found that *S. cerevisiae*, which shows the overflow phenomenon, has an increased metabolic flux span and an excess of available reducing capacity in all phenotypes where growth is limited by oxygen uptake. On the other hand, *S. stipitis* shows an almost invariant flux span and its available reducing capacity was found to be in excess only in a subset of the phenotypes limited by oxygen uptake. Although physiological features and metabolic routes other than ethanol production (e.g. polyols production in *S. stipitis*) have been described to account for the fermentative behavior of yeast, the approach presented in this work contributes to the characterization of yeast metabolism from the point of view of the entire flux network sensitive response, namely the available reducing capacity pattern. Therefore, a relationship between a property of the metabolic network: the shadow price, and a metabolic function: fermentative behavior, has been established.

## Methods

Genome-scale metabolic models [29] and constraint-based methods [30], [33] were used to perform a comparative analysis between *S. stipitis* and *S. cerevisiae*. Thus, a number of *in silico* analyses were carried out: ethanol-biomass production envelopes, sensitivity analysis regarding available reducing capacity, flux variability analysis and ethanol overproducing phenotypes calculation.

### Models implementation and FBA

Genome-scale metabolic models used in this work were: iMM904 [41] for *S. cerevisiae* and iBB814 [4] for *S. stipitis*. These models were chosen because they were constructed using experimental data, and have been empirically validated by a number of studies [28], [42]–[44]. Modeling approach considered extensive use of Flux Balance Analysis (FBA) [32]. Loop law constraints were added to all FBA calculations according to Schellenberg et al., [45] method so that infeasible loops were not allowed. Implementation of metabolic reconstructions and constrained-based analysis were done using COBRA Toolbox 2.0 [46] with MATLAB 2012a and TomLab/CPLEX v7.8 optimizer. MATLAB codes for all referenced COBRA functions are available at the COBRA's website (http://opencobra.sourceforge.net/).

### Production envelopes analysis

Production envelopes were determined using COBRA function productionEnvelope. Envelopes for ethanol production were calculated in two different ways: (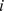) considering an unbounded oxygen uptake rate for *S. stitpitis* and (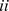) setting an upper bound for oxygen uptake rate (2.8 mmol gDW^−1^ h^−1^) for *S. cerevisiae*.

### Phenotypic phase planes analysis

Phenotypic phase planes (PhPPs) analysis [36] was performed characterizing all optimal flux distributions as a function of glucose and oxygen uptake rates. PhPPs were obtained by varying in a step wise fashion the two fluxes and calculating the optimal FBA objective value.

### Sensitivity analyses

Shadow price (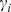, Eq. 1) corresponds to the sensitivity of the biomass flux as an objective function (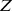) in response to a change in the availability of a metabolite (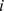), and indicates how much an increment in the availability of a metabolite will increase or decrease the growth rate [32]. Shadow price is defined as:
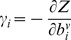
(1)where, 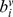 is equivalent to an exchange flux for the metabolite and defines the violation of the mass balance constraint [34], [36]. Therefore, shadow price captures the change in the value of the growth rate when one of the intracellular metabolites deviates from steady state [35].

The shadow price for each metabolite can be calculated as the solution vector of the corresponding dual problem of the FBA (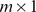 vector where 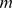 is the number of metabolites) [34]. In this work, this vector was automatically obtained from the dual problem implemented in TomLab/CPLEX via COBRA function optimizeCbModel. The sensitivity analysis was performed considering a shadow price for the available reducing capacity (Eq. 2) [36] which corresponds to the difference between the shadow price for NADH and NAD:
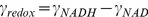
(2)


A negative value for 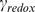 means that if available reducing capacity increases, growth rate also increases, indicating it is a growth-limiting metabolic resource. On the other hand, a positive value means that in order to improve growth rate, available reducing capacity should decrease, indicating that is available in excess for biomass production.

### Flux variability analysis (FVA)

In order to determine flux span, Flux Variability Analysis (FVA) [38] was performed by using COBRA function fluxVariability. The flux span (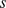) was calculated as shown in Eq. 3 where 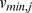 and 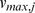 are the minimal and maximal 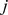 fluxes determined determined by the FVA.
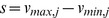
(3)


### Reaction deletion analysis

#### *A priori* approach

With the purpose of replicating in *S. stipitis* the 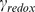 pattern of *S. cerevisiae*, flux value for certain reactions of the respiratory chain of *S. stipitis* were fixed to zero in the FBA to simulate metabolic deletions. These modifications were NDH1/AOX double-deletion and COX single-deletion.

#### Optimization-based approach

*In-silico* strains which enhance ethanol production were obtained via optimization-based approach by using Genetic Design through Local Search (GDLS) algorithm [47] (COBRA function GDLS). All solutions were checked to be unique and whether they were coupled or non-coupled to growth by using COBRA function analyseOptKnock, non-unique solutions were discarded. To obtain strains yielding different maximal specific growth rate, lower bound for growth rate was set in an increasing stepwise fashion from 0.1 to 0.7 h^−1^.

#### Modified model

Since an excess in available reducing capacity is searched for, it was attempted to force an increase in the available NADH. By means of adding an artificial sink reaction the metabolism was driven to function with a low availability of NADH. Then modifications (deletions) were sought in the direction of compensating for the NADH demanded by the sink. These modifications were searched for using a bilevel optimization aproach (GDLS), because it finds deletions leading to flux coupling, in this case ethanol to biomass production. The formulation of the problem using this approach considered the maximization of both ethanol and biomass fluxes. In this way, considering the modified model (with the NADH sink), the modifications are conditioned not only by the metabolic NADH demand for growth and ethanol, but also for the sink. Thus, an increment in the available NADH is expected by applying those modifications to the original model. This strategy was used with the purpose of finding more deletions leading *S. stipitis* in-silico strains to show an excess in available reducing capacity, as found in *S. cerevisiae*. For the NADH sink reaction fluxes between 0.1 and 0.6 mmol gDW^−1^ h^−1^, and a minimal growth rate of 0.20 h^−1^ were considered. Strains with single deletions which maximize ethanol flux were obtained using the modified model. The obtained deletions configured phenotypes with enhanced ethanol flux compensating for the low NADH availability generated by the sink. These single reaction deletions were applied to the non-modified model (the original one) enhancing ethanol flux and also increasing available reducing capacity.
